# Sigmoid sinus thrombosis presenting with posterior alexia in a patient with Behçet's disease and polycythaemia: a case report

**DOI:** 10.1186/1752-1947-2-175

**Published:** 2008-05-23

**Authors:** P Thomas, A Keightley, R Kamble, N Gunasekera, MR Johnson

**Affiliations:** 1 Department of Medicine, Heatherwood and Wexham Park NHS Hospitals Trust, Wexham Street, Slough, Berkshire SL2 4HL, UK; 2 Northwest Thames Foundation School, London Deanery, London, UK; 3 Imperial College School of Medicine, Charing Cross Hospital, London W6 8RF, UK

## Abstract

**Introduction:**

A 59-year-old Caucasian woman presented with an acute onset of alexia, noticed whilst driving. She described how while she could read car number plates, she had lost the ability to read and understand words on roadside advertisements and car window stickers.

**Case presentation:**

Neurological examination was unremarkable apart from the inability to read full words or sentences. Imaging of the brain, initially computed tomography, followed by magnetic resonance venography, confirmed a diagnosis of sigmoid sinus thrombosis with associated venous infarction. The patient's past medical history revealed that she had suffered an ischemic stroke and following investigation for this, had undergone a nephrectomy for renal cell carcinoma. This was complicated by postoperative deep venous thrombosis. She had a persistent polycythaemia that was managed with venesection, and recently she had been diagnosed with Behçet's disease. Prior to this presentation, she had recently stopped her prophylactic antiplatelet medication as she was due to undergo a total knee replacement for osteoarthritis. She was managed with weight-adjusted, low molecular weight heparin followed by oral anticoagulation, and made a good recovery from her symptoms.

**Conclusion:**

This case illustrates a classical neurological syndrome, highlights the importance of cerebral venous and sinus thrombosis as a cause of stroke, and the importance of remaining vigilant to a person's changing risk of venous thrombosis with evolving comorbidity.

## Introduction

Cerebral venous and sinus thrombosis (CVST) is an uncommon cause of stroke. Hypercoagulable states via multiple mechanisms including factor V Leiden and prothrombin mutations, increased levels of factor VIII, IX and XI, deficiencies of antithrombin III, proteins C and S as well as a variety of medical and surgical comorbidities may confer increased risk of CVST. Whilst single or multiple concomitant thrombophilic factors may increase the relative risk of CVST, its low prevalence makes it difficult to identify patients at high absolute risk who may benefit from prophylactic anticoagulation. We describe a patient with posterior alexia secondary to sigmoid sinus thrombosis who had multiple independent risk factors for venous thrombosis prior to this stroke.

## Case presentation

The patient is a 59-year-old, right-handed Caucasian woman who presented with progressive headache and an abrupt onset of alexia. She described how whilst driving she realised that although she could read the figures on car registration plates, she had lost the ability to understand words and sentences seen on window stickers or roadside advertisements. Other than this deficit, she had not noticed any alteration in her vision.

Her medical history revealed that the patient had suffered an ischaemic stroke 15 months previously, causing temporary weakness in the right hand. Subsequently, she was discovered to have erythrocytosis, with a haemoglobin level of 181 g/l. Imaging of her abdomen revealed a mass within the right kidney for which she underwent right total nephrectomy. The diagnosis of renal cell carcinoma was confirmed on histology, and there was no evidence of tumour spread beyond the kidney. During the postoperative period, she suffered a proximal lower limb deep venous thrombosis (DVT), which was managed with 6 months of anticoagulation with warfarin. Despite the nephrectomy, her erythrocytosis persisted, and a series of venesections was commenced. This succeeded in reducing her haematocrit to a consistently satisfactory level. Eight months later she developed orogenital ulceration, a recurrent follicular rash, and vague ocular complaints. Just prior to this admission, a diagnosis of Behçet's disease had been made, and a course of prednisolone was commenced, with resolution of these symptoms. The patient also suffered from osteoarthritis of the hips and knees, and was due to undergo bilateral total knee replacements. One week prior to her acute admission, she had attended a preoperative clinic and on advice had ceased to take her daily 75 mg dose of aspirin.

On examination, she was orientated in time, place and person, exhibited a Mini Mental State Examination of 10/10 and held a fluent conversion during consultation. General examination revealed evidence of resolving oral and genital ulceration and an excoriated, vesicular rash localised to her back and thighs. Systemic examination was unremarkable. Primary visual pathways were intact and she exhibited normal patterns of gait and movement. It was only on further perceptual testing that deficits were found. On assessment of reading, she was unable to read words (short or long) or sentences; however, when asked to spell out the words letter by letter she performed accurately. This was mirrored by a Snellen chart reading. Tests of associative agnosia showed accurate recognition and reproduction of shapes and patterns and interpretation of real-life objects, faces and colours. She was able to hold a fluent conversation and perform basic arithmetic satisfactorily.

Haematological investigations revealed a normal haemoglobin concentration (13.4 g/dl) and haematocrit, with a low red blood cell concentration of 6.2 × 10^12^/litre and a mean cell volume of 73 fl. She had a leucocytosis with a white cell count of 13.3 × 10^9^/litre (neutrophils 10.1 × 10^9^/litre), although reactive protein C and erythrocyte sedimentation rate were normal. There was no abnormality in clotting, renal or liver function tests. Serum immunoglobulin levels demonstrated a mild polyclonal rise in immunoglobulin M concentration, although electrophoresis was normal. A subsequent thrombophilia screen detected no abnormality in activated protein C resistance, antithrombin activity, protein C activity or free protein S; prothrombin 20210A gene variant, anticardiolipin antibody and lupus anticoagulant were not detected.

**Figure 1:**
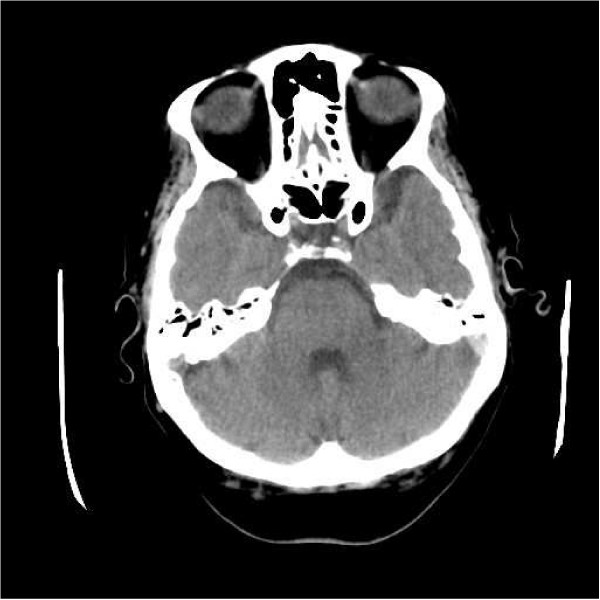
Axial section computed tomography scan demonstrating hyperdensity in the left sigmoid sinus.

**Figure 2:**
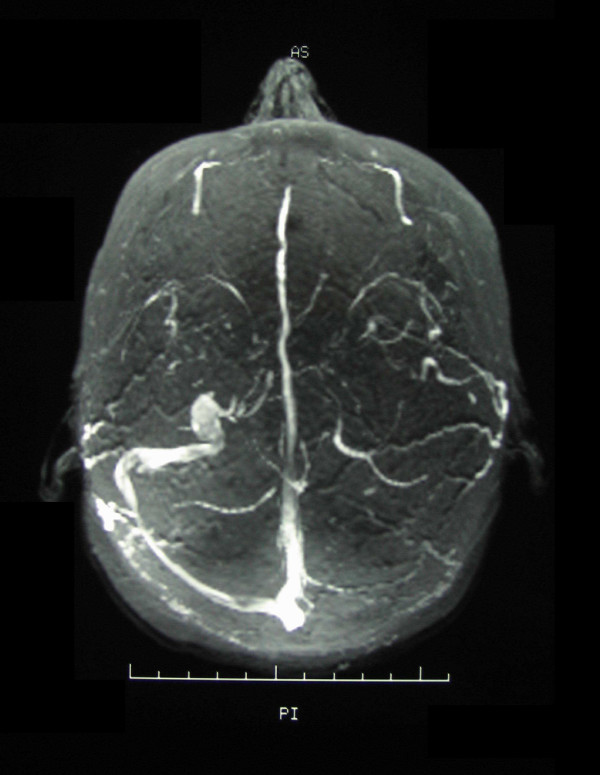
Magnetic resonance venogram demonstrating occlusion of the left sigmoid and transverse sinuses.

A clinical diagnosis of stroke involving the dominant (left) temporo-parietal region was made, and the patient underwent an emergency computed tomography (CT) scan of the head, with and without contrast. The left transverse and sigmoid sinuses were hyperdense, suggestive of thrombus (Figure 1) and there was a focal area of high attenuation in the left temporoparietal region, consistent with secondary haemorrhagic venous infarction. This was confirmed on magnetic resonance venography (Figure 2).

She was managed with body weight-adjusted, low molecular weight heparin for 2 weeks followed by oral anticoagulation. She made an excellent recovery over the course of this time with no formal rehabilitation, and at discharge had almost complete resolution of her symptoms, with no further problems apparent on follow-up examination.

## Discussion

Posterior (or pure) alexia is an uncommon acquired reading disturbance in which the ability to read is lost with maintenance of other language functions. It results as a consequence of disturbance of the visual temporo-occipital pathways and splenium of the corpus callosum [1]. The lesion almost always affects the dominant hemisphere, although it has been reported as a result of lesions in the nondominant side [2]. The pattern of symptoms is attributed to either disconnection of visual information to the language centre, or damage to a visual language association centre that resides in the affected area. Pathological processes are usually accompanied with a homonymous hemianopia owing to disturbance of the optic radiations; however, there is a smaller subset of patients who experience pure alexia without the presence of visual field defects. Posterior alexia is commonly thought to be word-length dependent; patients can often comprehend short words; however, complex multisyllable words evade the patient's perception.

Behçet's disease is a systemic vasculitis of unknown aetiology. Clinically, it is characterised by the presence of recurrent orogenital ulcers and uveitis, although other systemic manifestations are reported [3]. DVT is a recognised risk in patients with Behçet's, with a prevalence of 10% to 20% in case series, but incidence rates have not been determined in prospective studies [4]. The mechanism of venous thrombosis in Behçet's is unknown, and abnormal levels of thrombophilic factors do not appear to be responsible. In addition to Behçet's, our patient had two other conditions that increased her risk of venous thrombosis: polycythemia vera and a previous proximal lower limb DVT, although patients with postoperative venous thrombo-embolism are considered at very low risk of recurrence [5]. Polycythaemia vera may be complicated by both arterial and venous thrombosis and prospective studies have estimated the risk of cardiovascular death and nonfatal thrombotic events in polycythaemia vera to be 5.5 events/100 person years and aspirin has been shown to be effective in reducing this risk [6,7]. The likelihood of recurrent venous thrombosis is similar after CVST and lower extremity DVT at approximately 5.0/100 patient years [8].

The European Federation of Neurological Societies guidelines recommend that oral anticoagulation (INR 2–3) should be maintained indefinitely in patients with two or more episodes of CVST or one episode plus severe hereditary thrombophilia (that is, antithrombin deficiency, homozygous factor V Leiden mutation) [9]. Our patient does not have inherited thrombophilia, but does have medical comorbidities, including previous venous thrombo-embolism, polycythaemia and Behçet's that increase her future risk of CVST and so justify continued anticoagulation.

## Conclusion

This case report demonstrates an unusual presentation of a classical neurological disorder. For the junior clinician, an appreciation of the neuro-anatomical basis of the symptoms is necessary to form an accurate syndromic diagnosis. A key issue is the differentiation of cerebral venous infarction with secondary haemorrhage (an important issue in this case) from other causes of intracerebral haemorrhage including haemorrhagic transformation of ischaemic infarction or primary intracerebral haemorrhage: a cursory inspection of the CT may lead to misdiagnosis and subsequent mismanagement of the underlying cause of the stroke. In terms of the history and background of the case, we highlight the potential for people to develop a combination of thrombophilic states that act to increase significantly their risk of venous thrombosis. On review of the literature, we have found no reported cases with the same permutation of risk factors as our patient. It is difficult, therefore, to predict accurately her risk of thrombosis; however, these factors need to be appreciated in routine presurgical assessment clinics and at routine clinical review in order to optimally manage this risk. In our case, appropriate management might have involved the use of low molecular weight heparin perioperatively once aspirin therapy had been postponed.

## Abbreviations

CT: computed tomography; CVST: cerebral venous and sinus thrombosis; DVT: deep venous thrombosis.

## Competing interests

The authors declare that they have no competing interests.

## Consent

Written informed consent was obtained from the patient for publication of this case report and any accompanying images. A copy of the written consent is available for review by the Editor-in-Chief of this journal.

## Authors' contributions

PT, AK and RK were the junior doctors responsible for the acute and inpatient care of the patient, performed the research into the case and wrote the paper. NG and MRJ are consultant physicians who oversaw the care of the patient and advised and critically reviewed the content of the paper. All authors read and approved the final manuscript.
